# Very Stable High-Molecular-Mass Multiprotein Complexes in Different Organs of the Sea Cucumber *Paracaudina chilensis*

**DOI:** 10.3390/molecules30234496

**Published:** 2025-11-21

**Authors:** Svetlana E. Soboleva, Julia E. Poletaeva, Pavel S. Dmitrenok, Dmitrii V. Bulgakov, Elena I. Ryabchikova, Georgy A. Nevinsky

**Affiliations:** 1 Institute of Chemical Biology and Fundamental Medicine, Siberian Division of Russian Academy of Sciences, Pr. Akademika Lavrentieva, 8, Novosibirsk 630090, Russia; 2 G. B. Elyakov Pacific Institute of Bioorganic Chemistry, Far Eastern Branch of the Russian Academy of Sciences, 159 Pr. 100 let Vladivostoku, Vladivostok 690022, Russia; 3 Federal Scientific Center of the East Asia Terrestrial Biodiversity, Far Eastern Branch of the Russian Academy of Sciences, Vladivostok 690022, Russia

**Keywords:** sea cucumbers *Paracaudina chilensis*, stable multiprotein complexes, proteins, peptides

## Abstract

We report the first identification of several large (1.4–2.2 MDa), highly stable protein–peptide complexes in various organs and tissues (body wall, gonads, respiratory trees, gut, and coelomic fluid) of the sea cucumber *Paracaudina chilensis*. Gel filtration and transmission electron microscopy methods were used to estimate the molecular weights and sizes of the complexes. According to light scattering assay data, these multiprotein complexes undergo significant dissociation only in the presence of 3.0 M MgCl_2_ or 8.0 M urea containing 0.1 M EDTA and DTT. Analysis of the complexes using SDS-PAGE and MALDI mass spectrometry showed that all complexes contain numerous proteins (>10 kDa), whose number and composition vary among organs. Additionally, using MALDI mass spectrometry, it was shown that the whole-organism complexes contain 254 distinct peptides (<10 kDa). The peptide content in organ-specific complexes decreases in the following order: respiratory trees (104) > coelomic fluid (76) > body wall (64) > gut (58) > gonads (55). In contrast to individual proteins and peptides, multiprotein complexes have expanded possibilities, since they can interact with various molecules and cells. Thus, they can perform the functions of all peptides and proteins located on their surfaces. We propose that the unique protein and peptide composition of each complex facilitates the specific biological functions of its respective organ.

## 1. Introduction

It is known that a wide variety of biological processes are performed by different protein complexes [1]. For example, many biological processes require several proteins and enzymes, which are usually associated with each other, forming larger stable or temporary multiprotein complexes to significantly expand their biological functions, including increased efficiency of action, specificity, and the speed of metabolic pathways [2].

The most famous stable complexes are the large multi-subunit ribonucleoprotein complexes—ribosomes—which are necessary for protein synthesis [3]. It is interesting that ribosomes interact with various additional proteins and complexes to perform their functions. Myc and noncoding micro-RNA signaling pathways are found to be the primary mediators working jointly with RNA polymerases and ribosomal proteins to control ribosome biogenesis and protein synthesis [4]. Rapamycin complex 1 (mTORC1) is a key signaling complex leading to an expansion in translational capacity through ribosomal biogenesis, increased satellite cell abundance, and myonuclear accretion, as well as a post-exercise increase in muscle protein synthesis rates [4]. Three multiprotein complexes have key roles in transducing Wnt signals from the plasma membrane to the cell nucleus—the beta-catenin destruction complex, or axin degradasome; the Wnt signalosome, which involves polymerization of Dishevelled upon Wnt binding to its receptors to inactivate the Axin degradasome; and the Wnt enhanceosome, which enables β-catenin to gain access to target genes [5]. In Wnt signaling, an essential role of the Dishevelled DEP domain is shown. Limited dishevelled/Axin oligomerization determines the efficiency of Wnt/β-catenin signal transduction [6]. DEAD-box decapping enzyme 20 (DDX20) is a putative RNA-decapping enzyme that plays an important role in cellular transcription and post-transcriptional modifications [7]. The human GAIT complex of the translational control system is a heterotetramer containing glutamyl-prolyl tRNA synthetase (EPRS), NS1-associated protein (NSAP1), ribosomal protein L13a (L13a), and glyceraldehyde-3-phosphate dehydrogenase (GAPDH) [8]. Brain-derived neurotrophic factor (BDNF) is an essential regulator of synaptic transmission and long-term potentiation (LTP) in the hippocampus, as well as in other brain regions. The effects of BDNF in LTP are mediated by tropomyosin-related kinase B receptors, which are coupled with the activation of the phosphatidylinositol 3-kinase/Akt, phospholipase C-γ, and Ras/ERK signaling pathways [9].

Several other stable complexes have also been described. The Elg1 replication factor C-like multicomplex is essential for genome stability [10]. A special multi-subunit protein complex plays a fundamental role in eukaryotic mRNA metabolism and has a multitude of functions that impact eukaryotic gene expression [11]. Stable Polycomb multiprotein complexes are very important as chromatin regulators in eukaryotic gene transcription [12]. Several stable complexes important for quality control pathways of protein import into mitochondria have also been described [13]. Several specific complexes are also important for the functioning of signaling and regulatory proteins [14]. A multiprotein complex of the respiratory chain has been described [15]. Human membrane-associated complexes from the placenta, containing different proteins, were analyzed by SDS-PAGE and MALDI mass spectrometry; 34 novel heterooligomeric protein complexes were identified [16]. The authors of [17,18,19,20,21,22,23] discuss the basic physical–chemical principles underlying the formation of stable and unstable macromolecular complexes.

It can be assumed that there are many other multiprotein complexes in living organisms with a variety of biological functions that have not yet been discovered. Of particular interest may be those that are highly stable and specific to each organ. Numerous bioactive compounds are found in mother’s milk, placenta, and in the organs of various other organisms. The question arises as to which proteins and other components of biological fluids, cells, and organs can form temporary or stable complexes with different biological functions. Some complexes exhibit very high stability. It was recently shown that very stable multiprotein complexes (SPCs; ~1000 ± 100 kDa) exist in breast milk [24], human placentas [25,26], sea urchin eggs [27], and the sea cucumber *E. fraudatrix* [28]. These very stable protein complexes contain different proteins with low, moderate, and high molecular weights [24,25,26,27,28]. These complexes are stable in solutions containing 1.0–3.0 M NaCl, MgCl_2_, and 2.5–3.5 M urea and slowly dissociate only in the presence of 8.0 M urea containing 1.0 M NaCl and EDTA [24,25,26,27,28]. Very stable yet unknown multiprotein complexes are likely to exist in various biological fluids of different organisms. Thus, the formation of multiprotein complexes may lead to a vast expansion of their biological properties and functions, including their interactions with different proteins, nucleic acids, etc. Therefore, the search for and analysis of stable complexes with extended biological functions are of particular interest.

In this work, the isolation and analysis of new, very stable multiprotein complexes from the whole organisms of the sea cucumber *Paracaudina chilensis*, as well as from their individual organs and tissues (coelomic fluid, respiratory trees, gonads, gut, and body wall), were carried out for the first time. Widespread in the Sea of Japan, this species of the rat-tailed sea cucumber is practically unexplored and is known only for the presence of globins similar to human hemoglobin in its coelomic fluid [29]. Very interesting results were obtained. It turned out that all organs contain very stable protein complexes, but, depending on the organ, they differ in size, protein, and oligopeptide composition.

## 2. Results

### 2.1. Isolation of Protein Complexes from Sea Cucumbers

**Figure 1:**
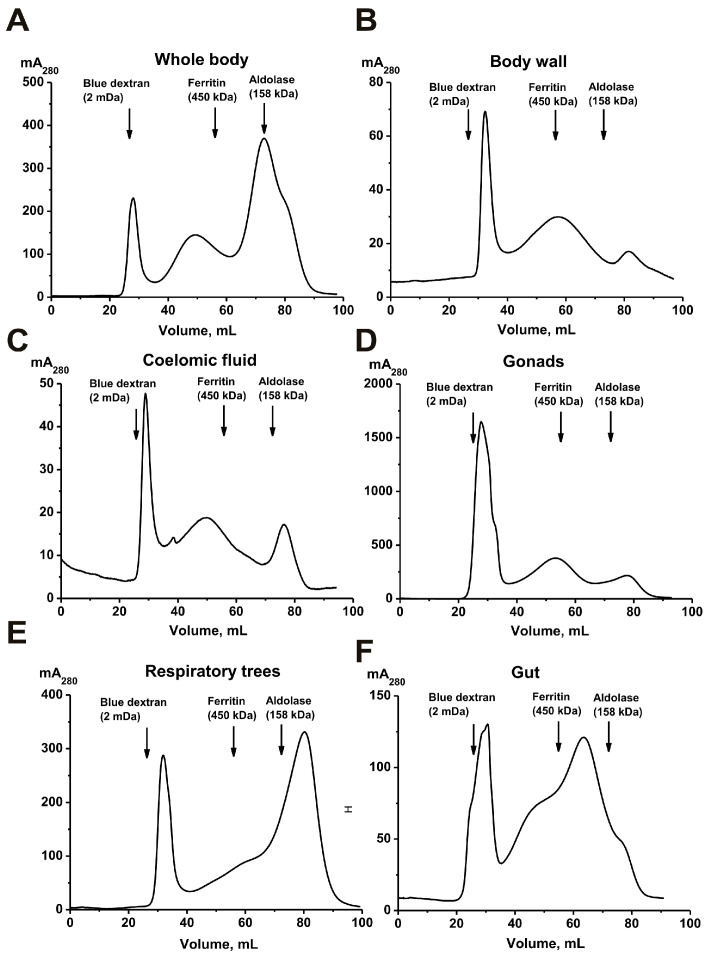
Isolation of protein complexes from the sea cucumber *P. chilensis* by FPLC gel filtration on a Sepharose 4B column using homogenates of intact sea cucumbers (**A**) and their different organs and tissues: body wall (**B**), coelomic fluid (**C**), gonads (**D**), respiratory trees (**E**), and gut (**F**).

Intact *P. chilensis* sea cucumbers and samples of their coelomic fluid (50 mL), as well as tissues from different body parts—(a) whole body, (b) body wall, (c) gonads, (d) respiratory trees, and (e) gut—were used. Mixtures of 5–7 intact cucumbers (100 g) and pooled samples of various organs and tissues from 30 to 40 sea cucumbers (10–20 g) were homogenized according to a method similar to [28]. All homogenates were concentrated and subjected to FPLC gel filtration on Sepharose 4B, which efficiently separates proteins with a molecular weight (MW) range of 50 to 20,000 kDa. Typical gel filtration profiles are shown in Figure 1.

All preparations were subjected to FPLC gel filtration under identical conditions. The first peaks correspond to high-molecular-weight protein complexes. In general, the gel filtration profiles are very similar in all cases and differ only in the relative content of protein material in the second and third peaks. However, the average molecular weights (MWs) of the complexes corresponding to the first peak after gel filtration differed slightly (mega Daltons) and were as follows: whole organism with all organs, 1.8 ± 0.1; gonads, 1.7 ± 0.2; body wall, 1.2 ± 0.2; coelomic fluid, 1.7 ± 0.2; respiratory trees, 1.4 ± 0.1; and gut, 1.5 ± 0.1. It was somewhat unexpected that the size of the complexes differed in different organs. Considering this, additional purification of the complexes from impurities of various proteins corresponding to the second peak was carried out using ultracentrifugation.

### 2.2. Transmission Electron Microscopy Analysis of Complexes

**Figure 2:**
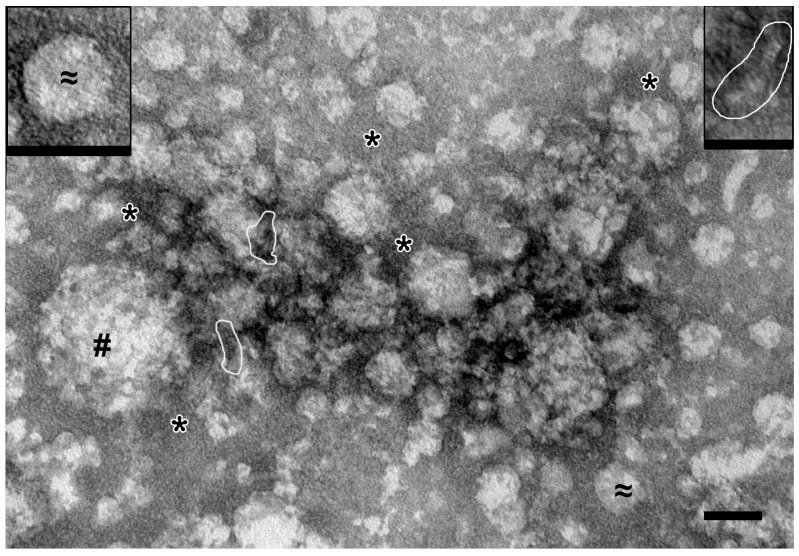
Representative image of complexes obtained by ultracentrifugation from full holothurian body including visceral organs. #—spherical particles of 40–140 nm (protein complexes); ≈ and insert—spherical particles of 30–40 nm (protein complexes); *—fine background material; stacks of “sticks” are shown by contour. TEM, negative staining by uranyl acetate. Length of scale bars corresponds to 50 nm.

All samples of the complexes obtained by ultracentrifugation were analyzed using transmission electron microscopy (TEM). Figure 2 shows the sample obtained from the entire holothurian body, including visceral organs.

Spherical particles with irregular contours and low electron density (40–140 nm in diameter) and spherical particles with smooth contours and medium electron density (30–40 nm) were observed. Fine-grained material and stacks of “sticks” and “dots” (referred to hereinafter as “elements”) were revealed between the spherical particles. Image analysis allowed us to conclude that the “sticks” may be constituent elements of spherical particles. Overall, it is clear that the preparation from the whole organism contains protein complexes of different sizes.

**Figure 3:**
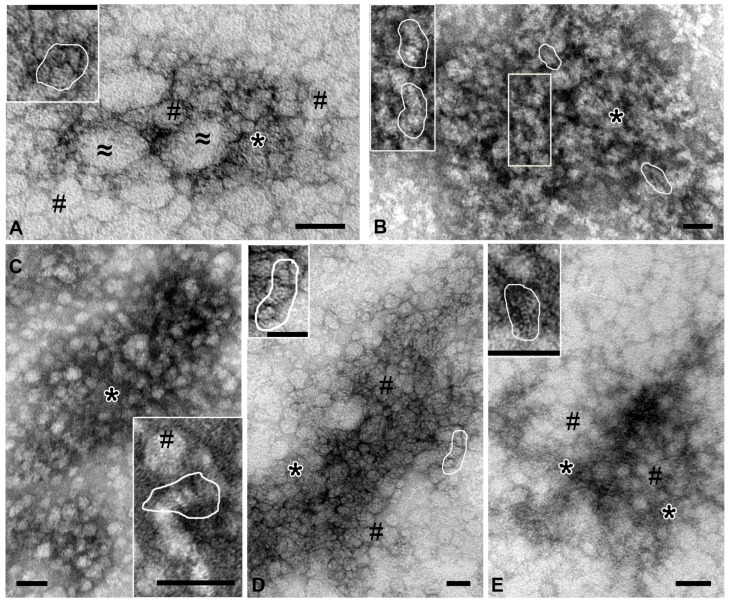
Representative images of complexes obtained by ultracentrifugation from different holothurian organs. (**A**)—coelomic fluid: #—spherical particles 20–30 nm; ≈—oval particles, up to 200 nm; *—an accumulation of small elements not organized into distinct structures. (**B**)—gut: stacks of “sticks” are shown in frames; *—accumulation of elements. (**C**)—respiratory trees: #—spherical particles; *—fine-grained material. (**D**)—gonads: #—spherical particles; *—accumulation of elements. (**E**)—body wall: #—spherical particles; *—fine-grained material. In all images, contours show stacks of “sticks”. Transmission electron microscopy, negative contrast. Length of scale bars corresponds to 50 nm.

In samples from the coelomic fluid (Figure 3A), spherical particles (20–30 nm) of low electron density with smooth contours were found; “sticks” and “dots” could be seen in their structure. Some particles were oval and reached 200 nm; their structure was poorly discernible. Between the particles there was material containing the elements. In the samples of high-molecular-weight complexes from the holothurian gut, the “sticks” are arranged in stacks 10–15 nm wide, which can be straight or curved (Figure 3B). Depending on the plane of sorption, the stacks form either bizarre patterns or chaotic accumulations in which individual elements are visible. Spherical particles were not found in the samples of gut complexes.

High-molecular-weight complexes isolated from the respiratory trees were uniformly adsorbed onto the grid, without forming dense accumulations. The “sticks” were arranged in stacks, which, in turn, could form spherical particles (up to 30 nm) of low electron density. Fine granular material filled the space between particles and stacks (Figure 3C).

The complex isolated from the gonads was represented by dense accumulations of particles of various shapes and sizes, which had an average electron density and clear contours (Figure 3D). “Sticks” and “dots” were visible in the structure of the particles or formed irregular accumulations. The body wall complex was adsorbed onto the grid as large arrays of spherical particles (~20 nm) of low electron density with fuzzy contours, which was due to the large amount of fine-grained material. Close inspection revealed that these particles contained stacks of “sticks” (Figure 3E, inset); “free” stacks were also observed between particles.

Analysis of the samples of complexes from different organs of holothurians revealed a variety of complex particles; however, all samples contained small “sticks” (2–3 nm). It can be assumed that the “sticks” represent the “basic” complex of holothurians. Gel filtration (Figure 1) and electron microscopy (Figure 3) data indicate that various organs of the sea cucumber contain protein complexes of different sizes.

### 2.3. Light Scattering Assay

**Figure 4:**
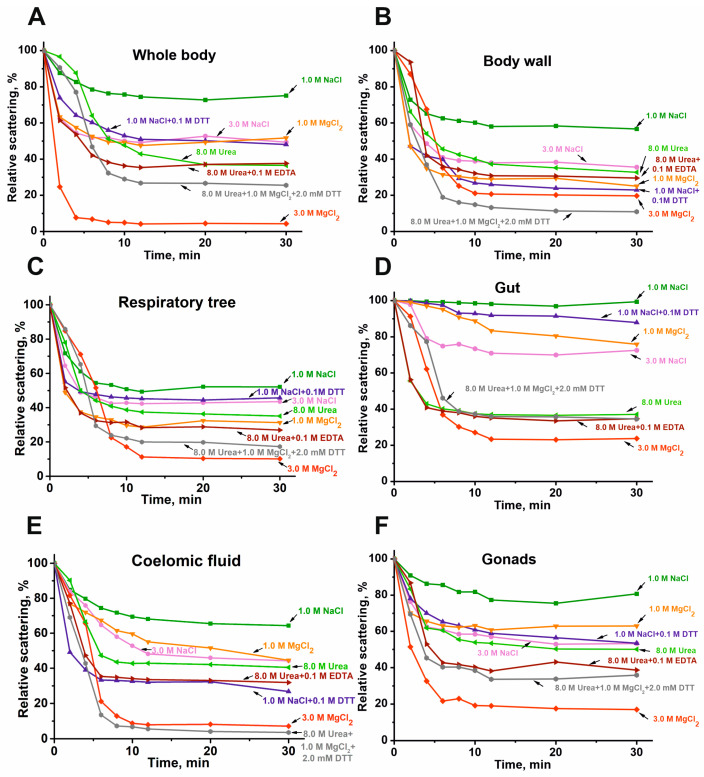
DLS data on typical examples of changes in the relative stability of the complexes (0.005–0.01 mg/mL) from the whole body (**A**), body wall (**B**), respiratory trees (**C**), gut (**D**), coelomic fluid (**E**), and gonads (**F**) in different conditions.

As shown earlier, all previously analyzed complexes from placenta [25,26], human milk [24], sea urchins [27], and the sea cucumber *E. fraudatrix* [28] are very stable and are destroyed only under very harsh conditions. The stability of the complex from the sea cucumber *P. chilensis* was analyzed by the dynamic light scattering (DLS) method (Figure 4).

Dissociation of all complexes is minimal in the presence of 1.0 M NaCl. The dissociation rate increases in the presence of 1.0 M MgCl_2_ or 3.0 M NaCl. The dissociation efficiency of all stable protein complexes is further enhanced in the presence of 8.0 M urea, and especially in a solution of 8.0 M urea, 1.0 M MgCl_2_, and 0.1 M EDTA, as well as in the presence of 1.0 M NaCl + 0.1 M DTT. The most effective disruption of all complexes is observed in the presence of 3.0 M MgCl_2_, a condition known to disrupt even very stable immune complexes. These data on the remarkably high and, to some extent, comparable stability of the protein complexes from the sea cucumber *P. chilensis* are in good agreement with the similar stability reported for multiprotein complexes from placenta [25,26], human milk [24], sea urchins [27], and the sea cucumber *E. fraudatrix* [28].

**Figure 5:**
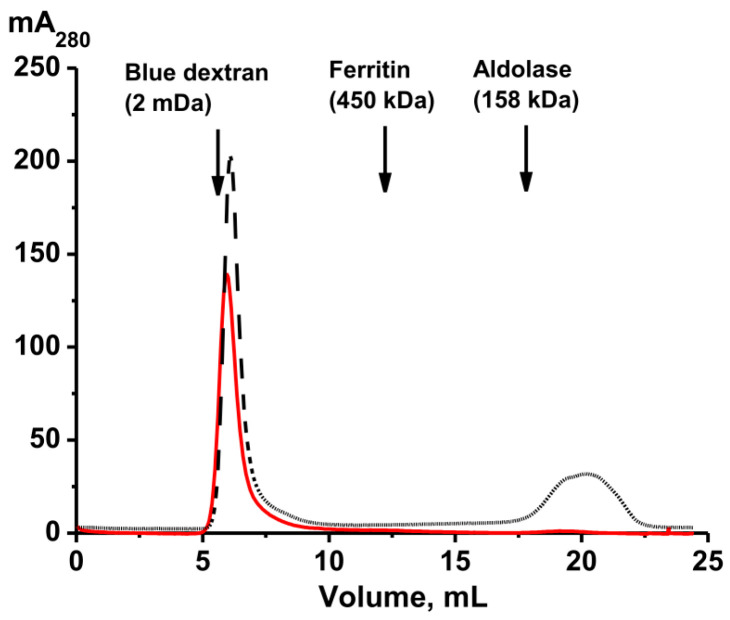
Gel filtration on Sepharose 4B of the whole-body complex before (red line) and after (black line) its treatment under severe conditions (8.0 M urea containing 1.0 M NaCl).

It can be assumed that, even under the most severe conditions (3.0 M MgCl_2_), only partial disruption of the complexes occurs—most likely the dissociation of components localized on the surface. Figure 5 shows the gel filtration profile of the gonad complex on Sepharose 4B after treatment under these denaturing conditions.

The top of the principal first peak (Figure 1) corresponding to the initial complex has shifted slightly compared to the peak before treatment, but peaks of proteins with lower molecular weights have appeared. Most likely, the complex of the core components of the original complex is even more stable. This is consistent with the data on the stability of other multiprotein complexes described earlier [24,25,26,27,28].

### 2.4. SDS-PAGE Assay of the Complex Proteins

**Figure 6:**
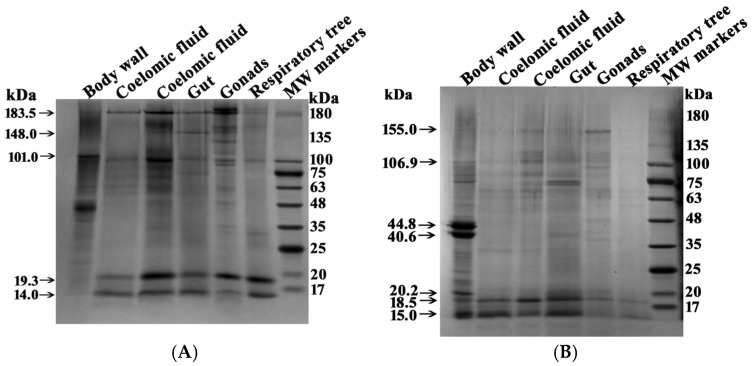
SDS-PAGE analysis of multiprotein complexes (4–16 µg) from different organs of sea cucumbers. Separation was performed on a 4–18% gradient gel before (**A**) and after (**B**) treatment with DTT. Molecular mass markers are indicated. The gels were stained using Coomassie R-250. For other details, see Section 4.

Proteins from the complexes corresponding to different organs were analyzed using SDS-PAGE (Figure 6).

Several major proteins were detected in each complex before treatment with DTT. However, the five complexes from different organs showed distinct protein profiles (Table 1). After treatment with DTT, the number of protein bands corresponding to high-molecular-weight proteins changed significantly (Figure 6, Table 1 and Table 2). This indicates that some of the protein bands before treatment of the complexes with DTT correspond to small protein fragments of complexes in which proteins are linked by S-S disulfide bonds.

Five protein complexes before treatment with DTT contain from 6 to 15 major bands corresponding to proteins or their complexes. Six major protein bands were found in the complex from the respiratory trees, and fifteen were found in the complex from the gonads. Only two protein bands (183.5 and 101.0 kDa) were common to all five complexes. Several proteins were found in four complexes, while others were present in only three or two. Proteins, or probably their complexes, with MWs 134.2 ± 8.8 and 35.4 ± 1.7 kDa were found only in the stable complexes from the gonads.

In general, the protein compositions of the five multiprotein complexes from different organs differed substantially. Thus, complexes from different organs vary not only in size (Figure 1 and Figure 3) but also in their protein composition. Interestingly, after treatment with DTT, proteins of 15.0 and 18.5 kDa were present in all complexes in varying amounts. In the body wall complex, the major proteins at 46.2 and 39.9 kDa were observed both before and after DTT treatment (Figure 6, Table 1). The intensity of some protein bands was significantly reduced after DTT treatment. Consequently, it is difficult to determine whether these bands represent incompletely dissociated small complexes present before DTT treatment (Figure 6A) or individual minor proteins from these complexes.

Figure 6 shows the SDS-PAGE analysis of the body wall complex, which contains prominent proteins at 46.2 and 39.9 kDa. Additionally, major proteins of approximately 19 and 14 kDa were detected in all complexes. These proteins were identified using MS and MS/MS analysis of their tryptic hydrolysates by MALDI mass spectrometry. The 46.2 and 39.9 kDa proteins were identified as actin and tropomyosin, respectively. The 19.3 kDa protein was identified as globin-1. The ~14.0 kDa band contained two co-migrating proteins: fragments of globin-1 and 12-oxophytodienoate reductase.

Overall, it appears that different forms of globin-1 with molecular masses in the 14.0–15.0 kDa and ~19 kDa ranges may be particularly important for the assembly of intact complexes across all organs.

### 2.5. MALDI Mass Analysis of Proteins

**Figure 7:**
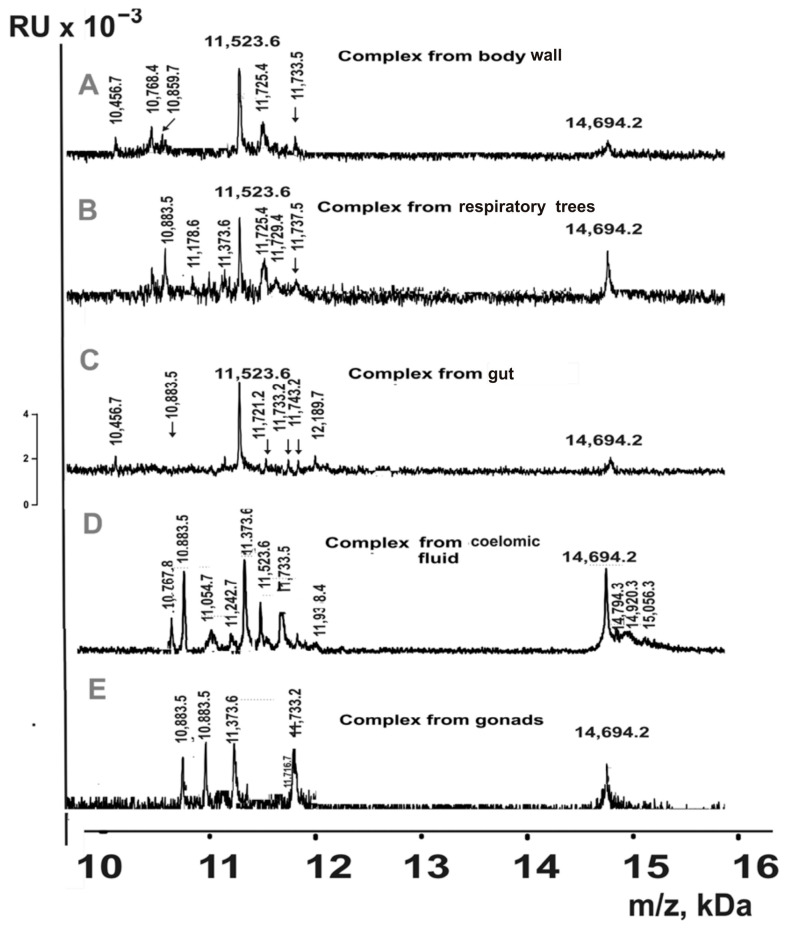
MALDI spectra of 10–20 kDa proteins eluted from Tips C18 by acetonitrile for complexes from different organs of sea cucumbers: body wall ((**A**); 20% acetonitrile), respiratory trees (**B**); 40%), gut (**C**); 70%), coelomic fluid (**D**); 30%), and gonads (**E**); 50%).

The SDS-PAGE approach allows for the determination of molecular weights of only the major proteins. Additionally, SDS-PAGE analysis does not allow for the separation of proteins with close molecular weights. Considering this, we analyzed proteins with molecular weights of 10–20 kDa using MALDI mass spectrometry. For this purpose, the complexes from different organs were first desalted and concentrated using ZIPTip Pipette Tips C18 for reversed-phase chromatography. The protein components of the complexes were eluted from the sorbent with acetonitrile gradients (5, 10, 20, 30, 40, 50, 70, 80, and 90%). The resulting fractions for all stable complexes were analyzed by MALDI mass spectrometry in the 10–20 kDa mass range. For each fraction eluted from the sorbent, 7–10 independent spectra were obtained. As an example, Figure 7 shows the spectra of some fractions.

Based on the analysis of the totality of all fractions’ spectra, the MWs of proteins eluted using 5–90% acetonitrile in the analyzed complexes were estimated (Table 2).

MALDI mass spectrometry detected 28 different proteins in the 10–20 kDa range across the set of multiprotein complexes from the whole organism of sea cucumbers (Table 2). Similarly to the SDS-PAGE analysis, proteins with molecular masses of 18.5 kDa and in the 14.8–15.0 kDa range were identified in complexes from all organs. The number of 10–20 kDa proteins varied among complexes from the five organs: coelomic fluid (15) > respiratory trees (12) = gut (12) > body wall (11) > gonads (7). Three proteins with molecular masses of 14,694.2 Da, 11,733.5 Da, and 10,883.5 Da were detected in complexes from all five organs. In addition to proteins common to multiple complexes, each complex contained unique proteins. It should be noted that some proteins had very similar molecular masses; however, Table 2 lists only those that corresponded to distinct peaks in the same spectrum. Nevertheless, it cannot be excluded that some proteins with closely matching masses represent the same protein with minor modifications.

Overall, these findings suggest that proteins of approximately 15.0 kDa and 18.5 kDa may be particularly important for the formation of complete complexes in all organs.

### 2.6. Peptides of the Complex

**Figure 8:**
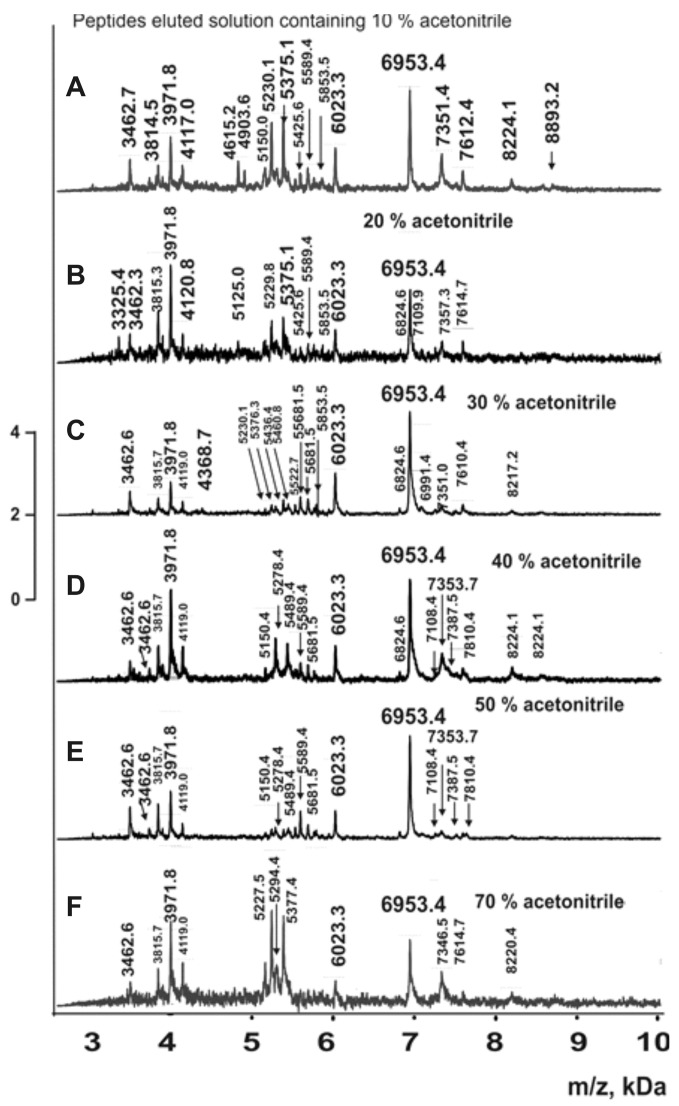
MALDI spectra of oligopeptides 2.5–10 kDa eluted from Tips C18 by different concentrations of acetonitrile for complexes from coelomic fluid (**A**–**F**).

As previously shown, multiprotein complexes from human milk, placenta, and sea urchin eggs contain numerous peptides with molecular masses <10 kDa in addition to proteins >10 kDa [24,25,26,27,28]. The analysis of complex mixtures containing multiple peptides and proteins presents a significant challenge. If any peptide co-crystallizes efficiently with the matrix, it suppresses the detection of other peptides. However, peptides <10 kDa can be relatively easily analyzed using the α-cyano-4-hydroxycinnamic acid (HCCA) matrix [30,31,32]. Initially, we analyzed the peptides in six complexes by applying aliquots of these complexes’ solutions to the iron target for MALDI analysis. The spectra contained numerous oligopeptide (OP) peaks <10 kDa, but with poor resolution. Therefore, for more detailed OP analysis, we performed preliminary separation using C18 reversed-phase chromatography (ZIPTip Pipette Tips). OPs were eluted with acetonitrile gradients (5–90%), and the resulting fractions from all stable complexes were analyzed by MALDI mass spectrometry in the 2–10 kDa mass range. For each fraction, 7–10 independent spectra were acquired. Representative spectra from different eluates of the coelomic fluid complex are shown in Figure 8.

Similar spectra were obtained for complexes from the whole organism, body wall, respiratory trees, gut, and gonads. In all cases, the molecular masses of all peptides in fractions eluted with 5–90% acetonitrile were determined. When molecular mass values for peptides were very close, they were considered reliable only if they corresponded to distinct peaks in the same spectrum. The average molecular mass values for each peptide were calculated using 7–10 spectra for each fraction eluted with acetonitrile solutions (5–90%). The fractions corresponding to the complex from the whole organism (all organs) contained 254 peptides with molecular masses ranging from 3 to 8.9 kDa (Supplementary Table S1). Complexes from individual organs contained fewer oligopeptides: respiratory trees (104) > coelomic fluid (76) > body wall (64) > gut (58) > gonads (55) (Supplementary Table S2). Complexes from all organs contained only five common major peptides: 6953.4; 5757.5; 5227.5; 3971.8; and 3814.5 Da (Table S2). Some oligopeptides were found in four or three complexes, while each complex contained several unique peptides not detected in other complexes (Table S2).

Currently, there are no data on multiprotein complexes from various organs of humans and other mammals. However, it is possible that in various organisms, multiprotein complexes may differ in composition and biological properties depending on the organ.

## 3. Discussion

Previous analyses have characterized multiprotein complexes from human placenta [25,26], milk [24], sea urchin [27], and the sea cucumber *E. fraudatrix* [28]. This study presents the first analysis of highly stable multiprotein complexes from five different organs of the sea cucumber *P. chilensis*. According to gel filtration data, complexes from different organs exhibit significant differences in their average molecular weights. The elution profiles of protein complexes from different organs are somewhat similar but show notable differences in the peak heights corresponding to lower-molecular-weight fractions following the principal complex peak (Figure 1). All five complexes demonstrated high stability, undergoing significant dissociation only under denaturing conditions comparable to those required for disrupting immune complexes (Figure 4). These findings are consistent with the high stability reported for multiprotein complexes from human milk, placenta, sea urchin, and *E. fraudatrix* [24,25,26,27,28]. Notably, maximum dissociation efficiency was observed in the presence of 3.0 M MgCl_2_, which primarily disrupts electrostatic interactions (Figure 4). However, substantial dissociation also occurred in 8.0 M urea, which mainly disrupts hydrogen bonds. These data indicate that both electrostatic contacts and hydrogen bonds are formed between the proteins and peptides of stable complexes (as in the case of complexes from milk, placenta, sea urchins, sea cucumber *E. fraudatrix* [24,25,26,27,28]). A noticeable increase in the rate of dissociation of the complexes after the addition of DTT (Figure 4) may indicate the formation of covalent disulfide bonds between the protein molecules.

Unexpectedly, electron microscopy revealed that the whole-body extract of the sea cucumber contains numerous protein complexes ranging in size from 10 to 100 nm (Figure 2). Analysis of complexes from different organs showed significant variations in size of protein complexes among the five organs (Figure 3), consistent with the gel filtration data. Further evidence for substantial differences among complexes from different holothurian organs was provided by SDS-PAGE (Figure 6). The five complexes contained different numbers of major proteins—from 6 to 15 (Table 1)—with no two complexes sharing an identical protein profile.

MALDI mass spectrometric analysis of reverse-phase column eluates from the whole-organism complexes revealed 28 proteins with molecular masses of 10–20 kDa (Table 2). However, the five complexes from individual organs differed in their content of these proteins. Even more notably, 254 oligopeptides with molecular masses of 3–10 kDa were identified in the whole-organism complex (Supplementary Table S1). Meanwhile, complexes from different organs contained distinct sets of peptides (Supplementary Table S2), with only a few peptides common to complexes from all five organs. Each complex contained unique, organ-specific oligopeptides (Table S2). Thus, stable complexes from different organs differ in both proteins (>10 kDa) and oligopeptides (<10 kDa). The formation of these highly stable complexes may be attributed to the intrinsic propensity of certain proteins and peptides for self-association [33,34,35,36,37].

Using SDS-PAGE and MALDI mass spectrometry analysis, proteins of 46.2 and 39.9 kDa were found, which were identified as actin and tropomyosin, respectively. Actin forms the cellular cytoskeleton, providing mechanical support. It participates in myosin-independent cell shape changes and cell movement and, together with myosin, forms a complex involved in muscle contraction. In non-muscle cells, actin participates in the transport of vesicles and organelles by myosin [38]. Tropomyosin can be found in various organs and body systems. In animals, it is an important component of the muscular system which works in conjunction with triponin to regulate muscle contraction [39].

Additionally, major proteins of approximately 19.3 kDa were identified as globin-1. The globins are a superfamily of heme-containing globular proteins, involved in binding and/or transporting oxygen [40]. Globin1 is required for the development and maintenance of the nervous system in Drosophila [41]. The ~14.0 kDa band contained two co-migrating proteins: fragments of globin-1 and 12-oxophytodienoate reductase. 12-oxophytodienoate reductase is an enzyme that utilizes the cofactor flavin mononucleotide and catalyzes the reaction of the jasmonic acid synthesis pathway [42].

As noted previously, multiprotein complexes—unlike individual proteins—possess an expanded capacity for interactions with various molecules in biological fluids and cells, as they can utilize the combined functional properties of all molecules present on their surface. Cells and biological fluids of different organisms contain components specific to each organ and fluid type, which are essential for carrying out organ-specific functions. Consequently, it can be proposed that multiprotein complexes located in different biological fluids and cells are likely to be distinct.

In this study, we determined the mass and protein composition of the complexes; however, their three-dimensional structures and biological functions remain unknown. Elucidating these aspects undoubtedly represents a significant goal for future research, as determining the native structure of macromolecules in solution is a challenging task. Techniques such as Small-Angle X-Ray Scattering (SAXS) and X-ray crystallography would be particularly valuable for this purpose. Furthermore, the biological functions of these complexes and their specific roles within the organism remain to be investigated.

## 4. Materials and Methods

### 4.1. Reagents

Adult *P. chilensis* sea cucumbers were collected from Peter the Great Bay in the Sea of Japan. These sea cucumbers are primarily found on the seafloor 7–15 m below the surface. The average salinity is approximately 34.5‰ (ppm) (https://oceanography-danchenkov.ru/oceanography/peter-the-great-bay-introduction/, 7 June 2025). The pressure at a depth of 7–15 m varies from 68,600 to 147,000 Pascals, and the temperature is approximately 7–0 °C in summer and 3–5 °C in winter.

Coelomic fluid and four organs (body wall, gut, respiratory trees, and gonads) were isolated from sea cucumbers before they were frozen. Then, all samples of holothurian organs were frozen and stored at −40 °C until the experiments. High-purity reagents (NaCl, SDS, Tris, EDTA, Coomassie blue, glycerol, and some other compounds) were from Sigma (St. Louis, MO, USA). The Sepharose 4B column was from GE Healthcare Life Sciences (New York, NY, USA). Reagents for EM studies were purchased from EMS (Houston, TX, USA).

### 4.2. Obtaining Sea Cucumber Extracts 

To obtain different homogenates, we used samples from intact *P chilensis* sea cucumbers, including coelomic fluid (50 mL) and various organs: (a) the whole body, (b) body wall, (c) gonads, (d) respiratory trees, and (e) gut. Mixtures of 5–7 intact cucumbers (100 g) and pooled samples from various organs of 30–40 sea cucumbers (10–20 g) were used. Before lysis, all samples were washed three times with buffer A (10 mM Tris-HCl, pH 8.0, 0.1 M NaCl) containing antibiotics (10^4^ U/mL penicillin, 25 U/mL ampicillin, and 10^4^ U/mL streptomycin) to remove surface bacteria. For lysis, tissue fragments were mixed with buffer A supplemented with 1.0 mM DTT, 1.0 mM EDTA, and antibiotics in a 1:10 (*w*/*v*) ratio and homogenized. All samples were homogenized under identical conditions. The homogenates were centrifuged at 16,500× *g* for 40 min (Avanti J-E centrifuge; Beckman Coulter, Brea, CA, USA). The supernatants were sonicated for 10 min at 30% amplitude (QSonica Q125 sonicator, New Haven, CT, USA) to remove lipids and then dialyzed against 2.0 L of 10 mM Tris-HCl (pH 8.0) at 4 °C with two changes at 4 h intervals followed by overnight dialysis.

### 4.3. Complex Separation and Purification

It has previously been shown that intact *E. fraudatrix* sea cucumber extract contains a very stable high-molecular-weight multiprotein complex (~2.0 mDa) and weaker associates of different proteins [28]. Therefore, similar to [41], to remove possible stable protein complexes, the extract of intact *P. chilensis* sea cucumber was subjected to gel filtration on Sepharose 4B, efficiently separating various proteins with molecular weights (MWs) of 60–20,000 kDa. The concentrated protein preparations (1 mL) were applied on a column with Sepharose 4B (90 mL) equilibrated in TBS buffer (20 mM Tris HCl pH 7.5, 0.5 M NaCl, and all antibiotics) using a GE Akta Purifier chromatograph (Chicago, IL, USA), and fractions (4.0 mL) eluted by the same buffer were collected. The complex and other proteins were monitored by absorbance at 280 nm (A_280_). Isolation of stable complexes from various organs (body wall, gut, respiratory trees, and gonads) was carried out using the same conditions described above for the whole organism. To remove NaCl, all fractions were dialyzed against 10 mM Tris-HCl buffer (pH 7.5) at 4 °C overnight and subsequently concentrated using a rotary evaporator (Refrigerated Centri Vap Concentrator, Labconco, Kansas City, MO, USA). To search for the multiprotein complexes, all holothurian preparations corresponding to the first peak after gel filtration were used. Ultracentrifugation at 100,000× *g* for 2 h at a temperature of +4 °C was used to purify the preparations from various vesicles and cell membrane fragments (Optima XE-90 Ultracentrifuge; Beckman Coulter, CA, USA). Then, the supernatants were used for different types of analysis. All experiments were performed under sterile conditions.

### 4.4. Negative Staining for TEM Study

Each of the samples was applied for 1 min on a copper grid covered using a formvar film stabilized with carbon. Then, grids were placed on a drop of 0.5% water solution of uranyl acetate for 7–10 s. Then, liquids were removed using filter paper. All grids were analyzed in a transmission electron microscope Jem1400 (Jeol, Tokyo, Japan), and the images obtained were collected using a Veleta digital camera (EM SIS, Muenster, Germany). The measurements were made using ITEM software version 5.2 (EM SIS, Muenster, Germany).

### 4.5. SDS-PAGE Assay

Analysis of multiprotein complexes by SDS-PAGE was performed in a 5–18% gradient gel supplemented with 0.1% SDS, according to Laemmli. Before electrophoresis, protein samples (10–20 μg) were preincubated using buffer A containing 50 mM Tris-HCl (pH 6.8), 10% glycerol, 1% SDS, 0.025% bromophenol blue, and 10 mM EDTA for 8 min at 100 °C and then applied to the gel. Proteins were stained with colloid silver or Coomassie R-250. After staining the gels with Coomassie R-250, major proteins corresponding to complexes from different organs of the sea cucumber were detected. We used Image Lab 6.1 software to determine the molecular masses of proteins. Each analysis was performed at least three times, and average values were calculated for each protein.

### 4.6. MALDI Mass Spectrometry Analysis of Peptides and Proteins

Identification of major proteins of the complexes was carried out via MS and MS/MS data of MALDI-TOF mass analysis of their tryptic hydrolysates according to standard procedures described in [28]. All analyses of peptides and proteins were performed using the Reflex III system (Bruker Company; Frankfurt, Germany): 337 nm nitrogen laser VSL-337 ND, 3 ns pulse duration.

Protein identifications after SDS-PAGE were performed based on MS and MS/MS data. Protein identifications were accepted when they were established at a score greater than 40 and contained at least 3 peptides identified using the Mascot search engine. Unfortunately, there are no data on *P. chilensis* proteins in the literature. Therefore, the data were analyzed using the 2016 SwissProt program and UniProt (http://www.uniprot.org/uniprot/, 20 January 2025). A major peptide of the complex corresponding to the body wall with MWs according to SDS-PAGE data at ~46 kDa was identified as Actin-15A (Actin-15A OS = Mesocentrotus franciscanus OX = 1328066; nominal mass: 41,800 Da). A major protein at ~40 kDa was identified as Tropomyosin (Tropomyosin OS = Stichopus japonicus OX = 307972 GN = BSL78_18963; nominal mass: 46,820 Da).

Two major protenis with MWs 14.0–15.0 kDa and ~19 kDa were found after SDS-PAGE in the complex from the whole organism and five different organs (Figure 6B). A protein corresponding to ~19 kDa was identified as Globin-1 (Globin-1 OS = Paracaudina chilensis OX = 7700; nominal mass: 17,704 Da). In the protein spot corresponding to ~15.0, two proteins were revealed: a fragment of Globin-1 (OS = Paracaudina chilensis OX = 7700; nominal mass: 17,704 Da) and a fragment of 12-oxophytodienoate reductase 1 (OS = Auxenochlorella protothecoides OX = 3075; nominal mass: 39,716 Da).

The analysis of peptides and proteins of coelomic fluids and homogonezates of different organs was also performed using the Reflex III system (Bruker Company; Frankfurt, Germany): 337 nm nitrogen laser VSL-337 ND, 3 ns pulse duration.

Coelomic fluid or homogenates of different organs were first directly applied to an iron target for MALDI mass spectrometry, as in [30,31,32]. The spectra of proteins and peptides were poorly resolved, since the solutions and the complexes themselves contained metal ions and other components that reduced the quality of the spectra. To improve the MALDI mass spectra, the solutions of all 6 complexes (10 μL) were used for reverse-phase chromatography using ZIPTip Pipette Tips C18 (Sigma-Aldrich, St. Louis, MO, USA) according to a standard procedure. Different salts were removed by washing the column with 1 mL of water. Then, the proteins and peptides were eluted from the sorbent with several solutions (200 µL) containing acetonitrile in different concentrations: 5, 10, 20, 30, 40, 50, 70, 80, and 90% acetonitrile. To remove the acetonitrile, the resulting solutions were dried. The resulting preparations were dissolved in 10 µL of water. Aliquots of the resulting solutions (1–2 µL) were used for peptide and protein analysis using MALDI mass spectrometry, as described below.

To analyze all proteins (>10 kDa) or oligopeptides <10 kDa, 1–2 μL of solutions eluted from the sorbent was mixed with 1–2 μL of saturated solution of matrix solved in 0.1% acetonitrile and trifluoroacetic acid (1: 2). For assay of proteins (>10 kDa), the solutions (1–2 µL) were mixed with saturated solution of α-sinapinic acid (SA matrix), while for analysis of peptides, they were mixed with α-cyano-4-hydroxycinnamic acid (HCCA) matrix. Then, 1.5 μL of the mixtures obtained was applied on the MALDI steel plate, air-dried, and used for the MALDI analysis. All MALDI spectra were calibrated using standard peptides or protein mixtures II and I (Bruker Daltonic, Frankfurt, Germany) in the internal or external calibration mode. The analysis of peptide MWs before or after standard hydrolysis of proteins with trypsin was performed using Protein Calculator v3.3 (Scripps Research Institute; La Jolla, CA, USA).

## 5. Conclusions

We have identified several large (1.2–2.2 megadaltons), highly stable protein–peptide complexes in various organs (body wall, gonads, respiratory trees, gut, and coelomic fluid) of the sea cucumber *Paracaudina chilensis* for the first time. These complexes dissociate significantly only in the presence of 3.0 M MgCl_2_ or 8.0 M urea containing 0.1 M EDTA and DTT. All complexes contain numerous proteins with molecular weights >10 kDa, but the number and type of these proteins vary among organs. Additionally, the whole-organism complexes contained 254 different oligopeptides with molecular weights <10 kDa. However, the peptide composition differed among organ-specific complexes: respiratory trees (104) > coelomic fluid (76) > body wall (64) > gut (58) > gonads (55). Unlike individual proteins and peptides, multiprotein complexes can interact with various molecules and cells, thereby performing expanded functions through the multiple molecules presented on their surfaces. Cells and biological fluids contain organ-specific and fluid-specific components, suggesting that the unique composition of each complex may be important for executing organ-specific functions.

For a more complete understanding of biological functions of very stable complexes, it seems important to analyze their possible enzymatic functions and native three-dimensional structures including techniques such as Small-Angle X-Ray Scattering (SAXS) and X-ray crystallography.

## Figures and Tables

**Table 1:** Molecular masses of proteins in various complexes from different organs of *P. chilensis* determined from SDS-PAGE analysis data before treatment with DTT (kDa) *.

Body Wall	Coelomic Fluid	Gut	Gonads	Respiratory Trees
Number of Proteins				
10	11	7	15	6
**200.1 ± 13.7**	**200.1 ± 11.3**	-	**200.1 ± 12.0**	**200.1 ± 7.1**
183.5 ± 5.7 **	**183.5 ± 8.4**	**183.5 ± 8.2**	**183.5 ± 7.6**	**183.5 ± 19.5**
168.1 ± 6.4	162.5 ± 11.5	-	157.7 ± 10.3	-
-	**148.0 ± 12.9**	**148.0 ± 2.9**	**148.0 ± 9.6**	-
-	-	-	134.2 ± 8.8	-
108.8 ± 2.9	116.6 ± 7.2	-	114.3 ± 4.7	-
**101.0 ± 0.8**	**101.0 ± 1.9**	**101.0 ± 2.0**	**101.0 ± 2.8**	**101.0 ± 1.0**
-	84.8 ± 3.6	-	90.4 ± 3.3	-
-	71.3 ± 0.9	74.2 ± 1.4	-	-
**62.0 ± 0.1**	**62.0 ± 1.5**	**62.0 ± 2.5**	**62.0 ± 1.0**	-
46.2 ± 0.1	-	-	48.5 ± 1.4	-
39.9 ± 0.5	-	-	45.4 ± 0.1	
-	-		35.4 ± 1.7-	-
-	-	-	**32.6 ± 2.8**	**32.6 ± 0.2**
25.5 ± 0.4	-	-	-	26.3 ± 0.5
22.2 ± 0.9	-	-	-	-
-	**19.3 ± 0.1**	**19.3 ± 0.4**	**19.3 ± 0.6**	**19.3 ± 0.3**
	**14.0 ± 0.8**	**14.0 ± 0.8**	**14.0 ± 1.0**	**14.0 ± 0.7**

* Visually identical proteins in different complexes are highlighted in bold. ** Proteins that are found in all five complexes are marked in red. We used Image Lab 6.1 software to determine molecular masses from the SDS-PAGE data (for example, those in Figure 6). Each analysis was performed at least three times, and average values were calculated for each protein.

**Table 2:** Molecular masses (*m*/*z*) of 10–20 kDa proteins in stable multiprotein complexes from different organs of the sea cucumber *P. chilensis* (kDa).

	Whole Organism	Body Wall (11 Proteins)	Respiratory Trees (12 Proteins)	Gut (12 Proteins)	Coelomic Fluid (15 Proteins)	Gonads (7 Proteins)
1	18.566 ± 2.4	+ *	**+**	**+**	**+**	**+**
2	15.066 ± 2.4	**+**	**+**	**+**	**+**	**+**
3	14.920 ± 2.1	− *	**−**	**−**	**+**	**−**
4	14.794 ± 2.1	**−**	**−**	**−**	**+**	**−**
5	14.694.2 ± 2.1	**+**	**+**	**−**	**+**	**+**
6	13.559.0 ± 2.4	**−**	**−**	**−**	**+**	**−**
7	12.180.7 ± 2.8	**−**	**−**	**+**	**−**	**−**
8	11.743.6 ± 2.0	**−**	**−**	**+**	**−**	**−**
9	11.737.4 ± 1.7	**−**	**+**	**−**	**−**	**−**
10	11.733.5 ± 1.5	**+**	**−**	**+**	**+**	**+**
11	11.729.8 ± 2.5	**−**	**+**	**−**	**−**	**−**
12	11.725.7 ± 3.1	**+**	**+**	**−**	**−**	**−**
13	11.721.2 ± 2.2	**+**	**−**	**+**	**−**	**−**
14	11.716.1 ± 1.6	**−**	**−**	**+**	**+**	**+**
15	11.713.8 ± 2.3	**+**	**+**	**−**	**−**	**−**
16	11.660.3 ± 2.8	**−**	**−**	**+**	**−**	**−**
17	11.546.0 ± 1.9	**−**	**+**	**+**	**−**	**−**
18	11.526.0 ± 2.1	**−**	**+**	**+**	**−**	**−**
19	11.523.6 ± 1.8	**+**	**−**	**+**	**+**	**−**
20	11.393.6 ± 2.1	**−**	**−**	**−**	**+**	**−**
21	11.373.6 ± 1.6	**−**	**+**	**−**	**+**	**+**
22	11.242.7 ± 1.9	**−**	**−**	**−**	**+**	**−**
23	11.178.3 ± 2.2	**−**	**+**	**−**	**−**	**−**
24	11.054 ± 1.9	**−**	**−**	**−**	**+**	**−**
25	10.883.5 ± 1.2	**−**	**+**	**+**	**+**	**+**
26	10.859.6 ± 2.9	**+**	**−**	**−**	**−**	**−**
27	10.767.8 ± 2.4	**+**	**−**	**−**	**+**	**−**
28	10.456 ± 1.9	**+**	**−**	**−**	**−**	**−**

* + indicates the presence of this protein in the complex, − its absence.

## Data Availability

All data are given in the article.

## Supplementary Materials

The following supporting information can be downloaded at https://www.mdpi.com/article/10.3390/molecules30234496/s1: Table S1: Molecular masses (*m*/*z*) of peptides (<10 kDa) of multiprotein complexes of the whole body containing all organs of the sea cucumber *P. chilensis*; Table S2: Comparison of peptides of multiprotein complexes from different organs of the sea cucumber *P. chilensis*.
