# Supplementary material for: Very Stable High-Molecular-Mass Multiprotein Complexes in Different Organs of the Sea Cucumber Paracaudina chilensis
Source: Molecules. 2025 Nov 21;30(23):4496. doi: 10.3390/molecules30234496 (PMC12692967; doi:10.3390/molecules30234496)
Supplement: Supplementary file 1 [file molecules-30-04496-s001.zip › molecules-3958708-supplementary.pdf]

Supplementary Table S1 Molecular masses (m/z) of peptides (<10 kDa) of multiprotein complexes of whole body containing all organs of the see cucumber *P. chilensis*

Numbers of individual peptides and their MWs, Daltons					
**1**	8823.9	**87**	6982.3	**172**	5296.5
**2**	8805.3	**88**	6979.4	**173**	5297.2
**3**	8603.1	**89**	6975.4	**174**	5288.1
**4**	8597.3	**90**	6971.8	**175**	5287.5
**5**	8592.3	**91**	6955.9	**176**	5284.3
**6**	8570.9	**92**	6953.6	**177**	5276.9
**7**	8566.8	**93**	6951.5	**178**	5273.5
**8**	8564.9	**94**	6950.4	**179**	5263.7
**9**	8561.2	**95**	6928.7	**180**	5262.3
**10**	8556.4	**96**	6912.4	**181**	5253.9
**11**	8236.4	**97**	6903.6	**182**	*5245*
**12**	8229.0	**98**	6825.1	**183**	5235.3
**13**	8226.0	**99**	6821.2	**184**	5233.5
**14**	8213.5	***100***	6535.5	**185**	5232.6
**15**	8209.1	**101**	6112.3	**186**	5231.7
**16**	8201.7	**102**	6079.7	**187**	5230.7
**17**	8192.5	**103**	6078.3	**188**	5228.4
**18**	8023.5	**104**	6073.8	**189**	5226.4
**19**	7650.7	**105**	6059.4	**190**	5223.9
**20**	7645.2	**106**	6053.6	**191**	5215.5
**21**	7632.4	**107**	6049.4	**192**	5205.9
**22**	7617.8	**108**	6047.0	**193**	5189.8
**23**	7611.8	**109**	6043.6	**194**	5180.7
**24**	7610.1	**110**	6028.0	**195**	5170.5
**25**	7608.9	**111**	6026.6	**196**	5165.0
**26**	7607.6	**112**	6025.1	**197**	5155.9
**27**	7605.1	**113**	6024.5	**198**	5151.5
**28**	7600.5	**114**	6023.3	**199**	5148.6
**29**	7529.2	**115**	6021.9	**200**	5146.1
**30**	7527.4	**116**	5985.9	**201**	5139.8
**31**	7524.3	**117**	5944.7	**202**	5135.9
**32**	7518.5	**118**	5943.8	**203**	5131.9
**33**	7437.8	**119**	5942.6	**204**	5126.4
**34**	7421.9	**120**	5856.5	**205**	5122.5
**35**	7411.2	**121**	5791.7	**206**	5119.9
**36**	7383.5	**122**	5786.3	**207**	5077.8
**37**	7368.5	**123**	5785.9	**208**	5073.4
**38**	7358.8	**124**	5781.8	**209**	5070.8
**39**	7352.0	**125**	5779.0	**210**	5067.4
**40**	7350.9	**126**	5773.3	**211**	5063.1
**41**	7347.5	**127**	5761.6	**212**	5057.6
**42**	7345.9	**128**	5760.3	**213**	4811.0
**43**	7343.9	**129**	5758.2	**214**	4371.7
**44**	7341.5	**130**	5757.6	**215**	4369.7

**45**	7339.9	**131**	5754.7	**216**	4367.7
**46**	7331.8	**132**	5752.5	**217**	4347.0
**47**	7329.1	**133**	5706.7	**218**	4279.6
**48**	7326.9	**139**	5686.8	**219**	4130.2
**49**	7315.2	**134**	5684.3	**220**	4126.3
**50**	7313.9	**135**	5683.2	**221**	4128.4
**51**	7307.3	**136**	5682.5	**222**	4126.0
**52**	7286.1	**137**	5677.8	**223**	4122.3
**53**	7269.4	**138**	5628.7	**224**	4120.2
**54**	7266.0	**139**	5617.6	**225**	4119.2
**55**	7153.3	**140**	5609.8	**226**	4107.0
**56**	7118.8	**141**	5592.1	**227**	4013.8
**57**	7114.9	**142**	5590.5	**228**	4007.3
**58**	7111.1	**143**	5589.0	**229**	3998.7
**59**	7110.4	**144**	5588.1	**230**	3994.3
**60**	7094.9	**245**	5586.6	**231**	3982.5
**61**	7088.1	**146**	5527.5	**232**	3981.3
**62**	7084.4	**147**	5526.2	**233**	3979.4
**63**	7080.4	**148**	5525.5	**234**	3976.9
**64**	7072.0	**149**	5447.3	**235**	3971.4
**65**	7069.9	**150**	5440.1	**236**	3963.4
**66**	7062.9	**151**	5438.8	**237**	3873.3
**67**	7056.2	**152**	5433.3	**238**	3868.1
**68**	7047.8	**153**	5432.3	**239**	3863.6
**69**	7044.0	**154**	5431.6	**240**	3829.1
**70**	7039.6	**155**	5430.1	**241**	3826.5
**71**	7035.3	**156**	5410.3	**242**	3824.4
**72**	7031.3	**157**	5408.8	**243**	3823.0
**73**	7029.4	**158**	5403.0	**244**	3821.5
**74**	7026.9	**159**	5401.6	**245**	3815.4
**75**	7021.0	**160**	5397.6	**246**	3813.9
**76**	7017.9	**161**	5386.5	**247**	3794.3
**77**	7014.5	**162**	5382.2	**248**	3792.8
**78**	7012.3	**163**	5380.2	**249**	3706.7
**79**	7009.8	**164**	5378.5	**250**	3486.9
**80**	7001.6	**165**	5373.6	**251**	3482.4
**81**	6997.2	**166**	5324.6	**252**	3474.6
**82**	6994.6	**167**	5317.2	**253**	3472.7
**83**	6992.8	**168**	5305.8	**254**	3471.6
**84**	6989.3	**169**	5304.4		
**85**	6988.7	**170**	5299.3		
**86**	6986.7	**171**	5295.7		

*Errors in determined MWs varies from 0.5 to 1.0 Da. In the case of close values, they were considered reliable if they were determined in the same spectrum

Supplementary Table S2. Comparison of peptides of multuiprotein complexes from different organs of the see cucumber *P. chilensis*

Molecular weights of peptides in Daltons (*the assigned number for the peptide in particular organ****) ****						
**Respiratory trees (104 peptides)**	***Body wall*** **(64 peptides)**	***Gut*** **(58 peptides)**	**Coelomic fluid (76 peptides)**	**Gonads (55 peptides)**	**№**	**All peptides In all organs**
		8900.2 *(1)*			**1**	**8900.2**
8893.2 (1)					**2**	***8893.2***
		8399.4 *(2)*			**3**	**8399.4**
8224.1 (2)					**4**	***8224.1***
8220.4 *(3)*				8220.2 *(1)*	**5**	**8220.4**
8217.2 *(4)*			8217.2 *(1)*		**6**	**8217.2**
				8213.7 *(2)*	**7**	**8213.7**
		8197.8 *(3)*			**8**	**8197.8**
				8148.4 *(3)*	**9**	**8148.4**
	7905.8 *(1)*				**10**	**7905.8**
		7664.4 *(4)*	7664.4 *(2)*		**11**	**7664.4**
		7660.9 *(5)*			**12**	**7660.9**
		7637.9 *(6)*			**13**	**7637.9**
7620.5 *(5)*	7620.5 *(2)*	7620.5 *(7)*			**14**	**7620.5**
			7624.1 *(3)*		**15**	**7624.1**
			7616.5 *(4)*		**16**	**7616.5**
7614.7 *(6)*				7614.7 *(4)*	**17**	**7614.7**
7612.4 *(7)*		7612.4 *(8)*	7612.4 *(5)*		**18**	**7612.4**
7610.4 *(8)*			7610.4 *(6)*	7610.4 *(5)*	**19**	**7610.4**
		7606.4 *(9)*			**20**	**7610.4**
			7529.8 *(7)*		**21**	**7529.8**
	7453.3 *(3)*				**22**	**7453.3**
			7035.1 *(8)*		**23**	**7035.1**
7432.3 *(9)*					**24**	**7432.3**
7420.8 *(10)*		7020.5 *(10)*			**25**	**7420.8**
7413.9 *(11)*					**26**	**7413.9**
7406.0 *(12)*					**27**	**7406.0**
7387.5 *(13)*					**28**	**7387.5**
7365.2 *(14)*					**29**	**7365.2**
7 62.9 *(15)*				7362.9 *(6)*	**30**	**7 62.9**
	7358.3. *(4)*		7358.3 *(9)*		**31**	**7358.3**
7357.3 *(16)*		7357.3 *(11)*			**32**	**7357.3**
			7355.1 *(10)*		**33**	**7355.1**
7353.7 *(17)*		7353.7 *(12)*		7353.7 *(7)*	**34**	**7353.7**
7351.0 *(18)*	7351.0 *(5)*			7351.0 *(8)*	**35**	**7351.0**
7347.7 *(19)*		7347.7 *(13)*	7347.7 *(11)*	7348.7 *(9)*	**36**	**7347.7**
7345.6 *(20)*	7345.6 *(6)*	7345.6 *(14)*	7345.6 *(12)*		**37**	**7345.6**
		7342.3 *(15)*			**38**	**7342.3**
7337.8 *(21)*					**39**	**7337.8**
				7336.1 *(10)*	**40**	**7336.1**
		7332.1 *(16)*	7332.1 *(13)*		**41**	**7332.1**
		7326.5 *(17)*			**42**	**7326.5**
7108.9 *(22)*					**43**	**7108.9**
7099.5 *(23)*					**44**	**7099.5**
7091.3 *(24)*					**45**	**7091.3**
				7051.1 *(11)*	**46**	**7051.1**
		7043.9 *(18)*			**47**	**7043.9**
7035.7 *(25)*					**48**	**7035.7**
				7032.3 *(12)*	**49**	**7032.3**
7030.3 *(26)*					**50**	**7030.3**

			7025.5 *(14)*		**31**	**7025.5**
			703.8 *(15)*		**52**	**7023.8**
	7021.5 *(7)*	7021.5 *(19)*	7021.5 *(16)*		**53**	**7021.5**
7015.5 *(27)*		7015.5 *(20)*	7016.5 *(17)*	7015.5 *(13)*	**54**	**7015.5**
			7012.7 *(18)*		**55**	**7012.7**
	7010.5 *(8)*				**56**	**7010.5**
7006.7 *(28)*	7006.7 *(9)*	7006.7 *(21)*			**57**	**7006.7**
		7003.6 *(22)*			**58**	**7003.6**
			7002.8 *(19)*		**59**	**7002.8**
	7001.3 *(10)*				**60**	**7001.3**
6998.8 *(29)*					**61**	**6998.8**
	6995.3 *(11)*		6995.3 *(20)*	6995.3 *(14)*	**62**	**6995.3**
6991.7 *(30)*		6991.7 *(23)*			**63**	**6991.7**
			6989.2 *(21)*		**64**	**6989.2**
	6988.5 *(12)*		6988.5 *(22)*		**65**	**6988.5**
6977.7 *(31)*			6977.7 *(23)*		**66**	**6977.7**
			6976.5 *(24)*	6976.5 *(15)*	**67**	**6976.5**
6974.7 *(32)*	6974.7 *(13)*	6974.7 *(24)*			**68**	**6974.7**
6970.5 *(33)*		6970.5 *(25)*		6970.5 *(16)*	**69**	**6970.5**
6967.2 *(34)*					**70**	**6967.2**
				6965.6 *(17)*	**71**	**6965.6**
	6957.2 *(14)*				**72**	**6957.2**
6955.6 *(35)*			6955.6 *(25)*		**73**	**6955.6**
**6953.4 *(36)***	**6953.4 *(15)***	**6953.4 *(26)***	**6953.9 *(26)***	**6953.4 *(18)***	**74**	**6953.4**
6952.5 *(37)*			6952.5 *(27)*		**75**	**6952.5**
		6950.4 *(27)*			**76**	**6950.4**
				6946.2 *(19)*	**77**	**6946.2**
	6933.3 *(16)*				**78**	**6933.3**
			6926.3 *(28)*		**79**	**6926.3**
		6911.1 *(28)*			**80**	**6911.1**
			6828.0 *(29)*		**81**	**6828.0**
6824.6 *(38)*					**82**	**6824.6**
		6920.8 *(29)*			**83**	**6920.8**
		6826.4 *(30)*			**84**	**6826.4**
	6822.5 *(17)*				**85**	**6822.5**
6815.5 *(39)*					**86**	**6815.5**
	6602.8 *(18)*		6602.8 *(30)*		**87**	**6602.8**
			6521.9 *(31)*		**88**	**6521.9**
	6468.2 *(19)*				**89**	**6468.2**
6411.5 *(40)*					**90**	**6411.5**
6354.3 *(41)*					**91**	**6354.3**
		6289.2 *(31)*			**92**	**6289.2**
			6066.3 *(32)*		**93**	**6066.3**
6045.3 *(42)*			6045.3 *(33)*		**94**	**6045.3**
			6036.5 *(34)*		**95**	**6036.5**
			6028.4 *(35)*		**96**	**6028.4**
6025.0 *(43)*	6025.9 *(20)*		6025.9 *(36)*	6025.9 *(20)*	**97**	**6025.9**
				6024.3 *(21)*	**98**	**6024.3**
6023.3 *(44)*	6023.3 *(21)*		6023.3 *(37)*	6023.3 *(22)*	**99**	**6023.3 6**
6021.7 *(45)*		6021.7 *(32)*			**100**	**6021.7**
6019.9 *(46)*	6019.9 *(22)*				**101**	**6019.9**
		6018.2 *(33)*			**102**	**6019.9**
			6011.1 *(38)*		**103**	**6011.1**
				6001.6 *(23)*	**104**	**6001.6**
5853.5 *(47)*				5853.5 *(24)*	**105**	**5853.5**
	5805.6 *(23)*				**106**	**5805.6**

5786.3 *(48)*					**107**	**5786.3**
**5757.5 *(49)***	**5757.5 *(24)***	**5757.5 *(34)***	**5757.5 *(39)***	**5757.5 *(25)***	**108**	**5757.5**
	5754.5 *(25)*		5754.5 *(40)*		**109**	**5754.5**
	5707.3 *(26)*				**110**	**5707.3**
	5686.7 *(27)*	5686.7 *(35)*			**111**	**5686.7**
			5683.6 *(41)*		**112**	**5683.6**
			5681.5 *(42)*	5681.5 *(26)*	**113**	**5681.5**
			5680.0 *(43)*		**114**	**5680.0**
5589.4 *(51)*			5589.4 *(44)*	5589.4 *(27)*	**115**	**5589.4**
	5587.1 *(29)*		5587.1 *(45)*		**116**	**5587.1**
5582.2 *(52)*				5582.2 *(28)*	**117**	**5582.2**
				5676.6 *(29)*	**118**	**5676.6**
5526.4 *(53)*	5526.4 *(30)*			5526.4 *(30)*	**119**	**5526.4**
5523.7 *(54)*	5523.7 *(31)*		5523.7 *(46)*		**120**	**5523.7**
			5522.5 *(47)*		**121**	**5522.5**
5467.0 *(55)*					**122**	**5467.0**
5460.8 *(56)*					**123**	**5460.8**
5446.5 *(57)*					**124**	**5446.5**
5441.3 *(58)*					**125**	**5441.3**
			5435.6 *(48)*		**126**	**5435.6**
			5434.0 *(49)*	5434.0 *(31)*	**127**	**5434.0**
			5427.1 *(50)*		**128**	**5427.1**
5425.3 *(59)*					**129**	**5425.3**
	5423.3 *(32)*				**130**	**5423.3**
5436.6 *(60)*					**131**	**5436.6**
5418.7 *(61)*					**132**	**5418.7**
5403.7 *(62)*					**133**	**5403.7**
5400.3 *(63)*					**134**	**5400.3**
5394.0 *(64)*					**135**	**5394.0**
			5379.7 *(51)*		**136**	**5379.7**
5281.7 *(65)*	5379.7 *(33)*	5379.7 *(36)*			**137**	**5379.7**
5376.3 (66)	5376.3 *(34)*	5276.3 *(37)*			**138**	**5276.3**
		5375.1 *(38)*	5375.1 *(52)*	5375.1 *(32)*	**139**	**5375.1**
5373.6 *(67)*	5373.6 *(35)*			5373.6 *(33)*	**140**	**5373.6**
5306.0 *(68)*					**141**	**5306.0**
5302.3 *(69)*					**142**	**5302.3**
5298.9 *(70)*					**143**	**5298.9**
5294.0 *(71)*					**144**	**5294.0**
5284.3 *(72)*					**145**	**5284.3**
5278.7 *(73)*		5278.7 *(39)*			**146**	**5278.7**
5277.1 *(74)*			5277.1 *(53)*	5277.1 *(34)*	**147**	**5277.1**
	5268.5 *(36)*				**148**	**5268.5**
	5266.5 *(37)*				**149**	**5266.5**
5254.6 *(75)*					**150**	**5254.6**
5247.7 *(76)*					**151**	**5247.7**
5229.8 *(77)*	5229.8 *(38)*		5229.8 *(54)*	5229.8 *(35)*	**152**	**5229.8**
**5227.5 *(78)***	**5227.5 *(39)***	**5227.5 *(40)***	**5227.5 *(55)***	**5227.5 *(36)***	**153**	**5227.5**
5230.1 *(79)*					**154**	**5230.1**
5225.3 *(80)*		5225.3 *(41)*			**155**	**5225.3**
	5220.9 *(40)*				**156**	**5220.9**
			5215.7 *(56)*		**157**	
	5192.7 *(41)*				**158**	**5192.7**
	5183.7 *(42)*				**159**	**5183.7**
	5171.7 *(43)*				**160**	**5171.7**
	5164.9 *(44)*				**161**	**5164.9**
5150.0 *(81)*					**162**	**5150.0**
			5147.9 *(57)*	5147.9 *(37)*	**163**	**5147.9**

		5145.6 *(42)*			**164**	**5145.6**
				5135.7 *(38)*	**165**	**5135.7**
				5130.1 *(39)*	**166**	**5130.1**
	5098.2 *(45)*				**167**	**5098.2**
	5093.1 *(46)*				**168**	**5093.1**
		5045.2 *(43)*			**169**	**5045.2**
	4967.6 *(47)*				**170**	**4967.6**
		4925.7 *(44)*			**171**	**4925.7**
	4903.1 *(48)*				**172**	**4903.1**
	4833.1 *(49)*				**173**	**4833.1**
					**174**	
4815.2 *(82)*					**175**	**4815.2**
			4810.2 *(58)*		**176**	**4810.2**
	4669.4 *(50)*				**177**	**4669.4**
4573.7 *(83)*					**178**	**4573.7**
				4388.8 *(40)*	**179**	**4388.8**
	4368.7 *(51)*				**180**	**4368.7**
		4310.5 *(45)*			**181**	**4310.5**
		4293.0 *(46)*			**182**	**4293.0**
	4214.7 *(52)*				**183**	**4214.7**
	4206.2 *(53)*				**184**	**4206.2**
4147.0 *(84)*					**185**	**4147.0**
4142.8 *(85)*					**186**	**4142.8**
4120.8 *(86)*	4120.8 *(54)*		4120.8 *(59)*	4120.8 *(41)*	**187**	**4120.8**
4117.0 *(87)*					**188**	**4117.0**
	4055.5 *(55)*				**189**	**4055.5**
				4052.3 *(42)*	**190**	**4052.3**
4039.4 *(88)*			4039.4 *(60)*	4039.4 *(43)*	**191**	**4039.4**
				4035.3 *(44)*	**192**	**4035.3**
4033.6 *(89)*					**193**	**4033.6**
				4030.9 *(45)*	**194**	**4030.9**
4016.6 *(90)*					**195**	**4016.6**
				4014.7 *(46)*	**196**	**4014.7**
4012.4 *(91)*			4012.4 *(61)*		**197**	**4012.4**
				4010.7 *(47)*	**198**	**4010.7**
3997.3 *(92)*					**199**	**3997.3**
3995.3 *(93)*			3995.3 *(62)*		**200**	**3995.3**
			3994.6 *(63)*		**201**	**3994.6**
	3991.0 *(56)*				**202**	**3991.0**
	3977.9 *(57)*				**203**	**3977.9**
3973.4 *(94)*			3973.4 (64)		**204**	**3973.4**
**3971.8 *(95)***	**3971.8 *(58)***	**3971.8 *(47)***	**3971.8 *(65)***	**3971.8 *(48)***	**205**	**3971.8**
3962.8 *(96)*	3962.8 *(59)*				**206**	**3962.8**
	3960.1 *(60)*				**207**	**3960.1**
3878.3 *(97)*					**208**	**3878.3**
3874.4 *(98)*					**209**	**3874.4**
3871.9 *(99)*					**210**	**3871.9**
		3869.1 *(48)*			**211**	**3869.1**
			3841.4 *(66)*		**212**	**3841.4**
			3836.9 *(67)*		**213**	**3836.9**
		3829.4 *(49)*			**214**	**3713.1**
		3827.5 *(50)*			**215**	**3713.1**
				3825.7 *(49)*	**216**	**3825.7**
			3819.9 *(68)*		**217**	**3819.9**
			3817.0 *(69)*		**218**	**3817.0**
**3815.3 *(100)***	**3815.2 *(61)***	**3814.5 *(51)***	**3814.5 *(70)***	**3814.5 *(50)***	**219**	**3814.5**
			3812.4 *(71)*	3812.4 *(51)*	**220**	**3812.4**

		3798.6 *(52)*			**221**	**3798.6**
			3718.9 *(72)*		**222**	**3718.9**
	3713.1 *(62)*				**223**	**3713.1**
3711.6 *(101)*					**224**	**3711.6**
		3704.5 *(53)*			**225**	**3704.5**
		3692.2 *(54)*			**226**	**3692.2**
	3625.5 *(63)*				**227**	**3625.5**
	3524.4 *(64)*				**228**	**3524.4**
		3517.6 *(55)*			**229**	**3517.6**
3509.7 *(102)*					**230**	**3509.7**
3473.2 *(103)*		3473.2 *(56)*			**231**	**3473.2**
			3465.9 *(73)*	3465.9 *(52)*	**232**	**3465.9**
			3463.1 *(74)*	3463.1 *(53)*	**233**	**3463.1**
3462.6 *(104)*		3462.7 *(57)*	3462.7 *(75)*	3462.7 *(54)*	**234**	**3462.7**
			3455.7 *(76)*		**235**	**3455.7**
				3283.2 *(55)*	**236**	**3283.2**
		2996.5 *(58)*			**237**	**2996.5**

*Table S2 contains only those very close MWs, which corresponded to closely spaced peaks in the same spectrum. Five major peptides (numbers 74, 108, 153, 205, and 219) are highlighted in bold.
